# Efficacy and safety of renal denervation in patients with refractory ventricular electrical storm

**DOI:** 10.3389/fcvm.2026.1840013

**Published:** 2026-07-28

**Authors:** Guanghui Zhu, Xuelian Yuan, Yangpeng Li, Abubakari Xieherezhati, Lihua Zhao, Baopeng Tang, Yaodong Li

**Affiliations:** 1 Cardiac Pacing and Electrophysiology Department, The First Affiliated Hospital of Xinjiang Medical University, Urumqi, Xinjiang, China; 2 Xinjiang Key Laboratory of Cardiac Electrophysiology and Cardiac Remodeling, The First Affiliated Hospital of Xinjiang Medical University, Urumqi, Xinjiang, China

**Keywords:** implantable cardioverter defibrillator, radiofrequency ablation, renal denervation, ventricular arrhythmia (VA), ventricular electrical storm

## Abstract

**Background:**

Ventricular electrical storm (ES) is a malignant arrhythmia that seriously threatens the patients' life. Renal denervation (RDN) has been described as a treatment modality for refractory ventricular arrhythmias. This study aims to explore the potential role in patients with ablation-refractory ES.

**Methods:**

This single-center retrospective cohort study included patients with refractory ES who underwent RDN between 2023 and 2024 with a follow-up period for 2–6 months. Implantable cardioverter defibrillator (ICD) programming results and intraoperative parameters were collected. Patients' clinical examination results were also compiled.

**Results:**

The study involved 10 patients with a mean age of 57.9 ± 7.4 years, including 4 (40%) ischemic cardiomyopathy, 6 (60%) dilated cardiomyopathy and 6 (60%) hypertension. ICD therapies showed a decreasing trend after multimodal therapy including RDN [pre-RDN 22 [9.3, 41], post-RDN 2.5 [0, 26.3]]. ICD shock therapies exhibited a similar descending tendency [pre-RDN 8.5 [1, 12.8] and post-RDN 0.5 [0, 3.5]]. Echocardiography and plasma results remained unchanged before and after surgery, including ejection fraction, left ventricular end-systolic diameter, left ventricular end-diastolic diameter, and creatinine concentration.

**Conclusion:**

Multimodal therapy including RDN may has the potential to contribute to decrease ICD therapies for patients with ES. Larger sample investigations may further demonstrate the efficacy and safety of RDN.

## Introduction

The ventricular electrical storm (ES) is characterized by 3 or more instances of persistent ventricular arrhythmias (VAs) occurring within 24 h, separated by a minimum of 5 min, necessitating cessation through an intervention (1). Most patients with ES have underlying structural heart diseases (2). The overall mortality rate of ES is 2.5 times higher than that of sporadic VAs, which poses a serious risk to patients' lives (3). The current treatments for ES include medications, radiofrequency ablation (RFA), cardiac sympathetic denervation (CSD), and implantable cardioverter defibrillators (ICDs) (2, 4). Renal denervation (RDN) is an interventional procedure that emerged in recent years. It can inhibit the activation of the renin-angiotensin-aldosterone (RAS) system and decrease systemic sympathetic nerve activity. Initially, RDN was used to treat refractory hypertension and had made promising success, while accumulating evidence suggests that it might be beneficial to other diseases, such as heart failure (HF), arrhythmia, and cardiac hypertrophy (5–7).

Meanwhile, our center's previous animal experiments demonstrated the effectiveness of RDN in ventricular arrhythmias and cardiac reverse remodeling (8). Although safety and effectiveness evaluation of RDN in patients with VAs have been explored in previous studies, there are few studies on the treatment of refractory ES with RDN. So, the study further explores the effectiveness and safety of RDN in patients with ES. Since RFA is highly recommended as non-pharmacological treatment and long-term prevention strategy of ES, we meanwhile evaluate whether the timing of combined RDN and RFA impacts patient prognosis.

## Methods

### Study design and population

This is a retrospective, single-arm cohort study. Inclusion criteria: patients who underwent previous RFA with refractory ES (≥3 in 24 h) at our center from 2023 to 2024 were enrolled. Before RDN procedure, laboratory tests were performed to exclude reversible causes of ES, including electrolyte abnormalities, infection, thyroid dysfunction, and drug toxicity. Decompensated heart failure and ischemia was also assessed. Then baseline characteristics, indication for RDN, and available clinical 6-month follow-up were assessed. We quantified ICD therapies before and after RDN. Cardiac ultrasound and creatinine concentration were also assessed.

### Ethical approval

The study was conducted in accordance with the Declaration of Helsinki, and all patients provided written informed consent. Patients or the public were not involved in the design, or conduct, or reporting, or dissemination plans of our research. The study received approval from the Ethics Committee of the First Affiliated Hospital of Xinjiang Medical University (No. 210115-15-2501B-Y2).

### RDN procedure

**Figure 1:**
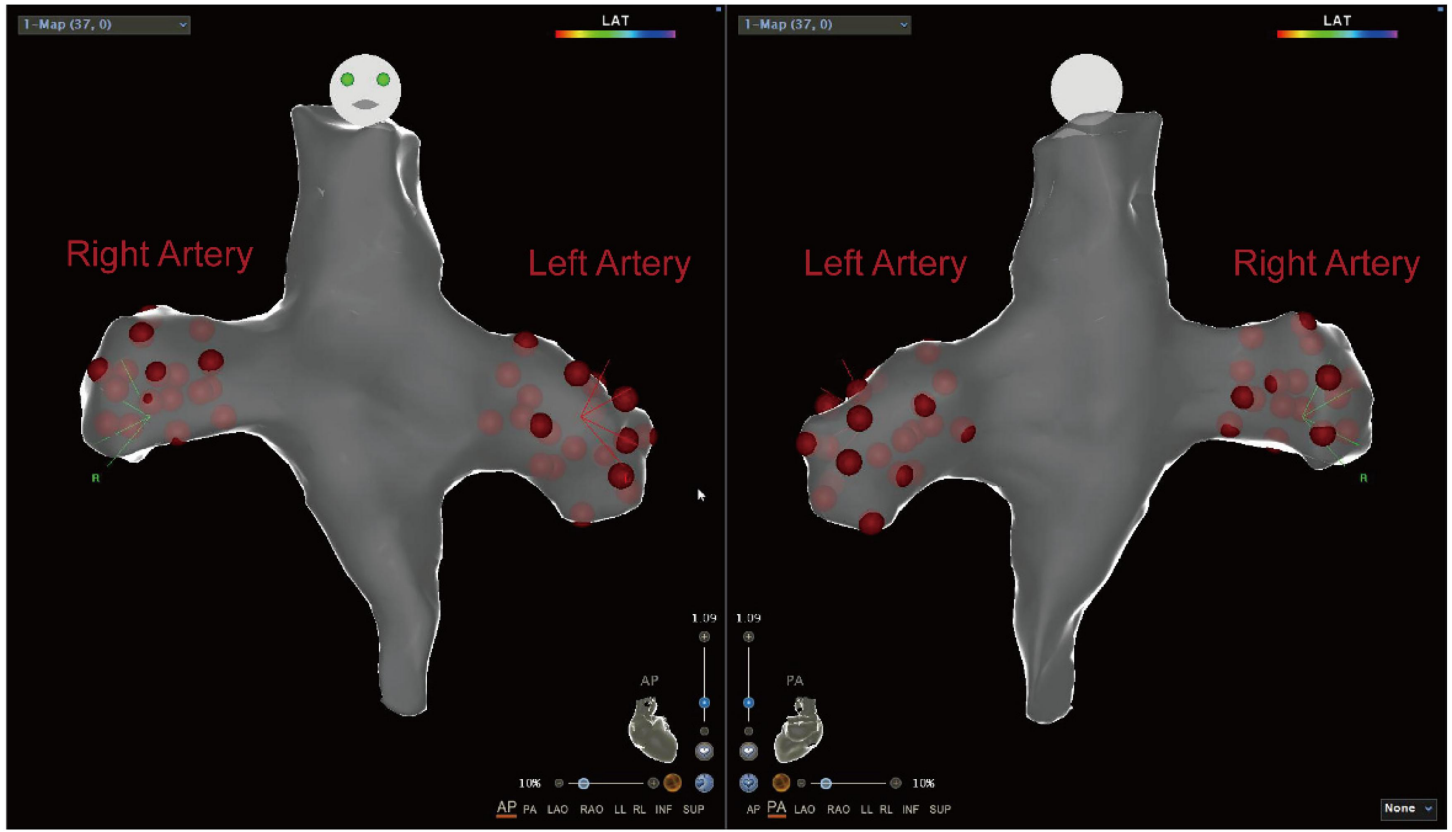
RDN modeling diagram. Each red dot represents an ablation lesion. The left image shows the anteroposterior view, and the right image shows the posteroanterior view; RDN, renal denervation.

With the patient in the supine position, an 8.5F sheath was inserted into the right femoral artery after local 2% lidocaine anesthesia. A JR4 catheter (Johnson & Johnson, USA) was advanced through the sheath to assess renal arteries. After withdrawing the JR4 catheter, an STSF catheter (Johnson & Johnson, USA) was advanced to both renal arteries. Three-dimensional reconstruction of the renal arteries was conducted by the CARTO3 mapping system (Johnson & Johnson, USA). Subsequently, point-by-point ablation was performed in a spiral pattern along the length of the renal artery, from the first distal bifurcation to its junction with the aorta, at an energy of 15 or 25 W. The duration of each point ablation was 20 or 30 s, and the distance between ablation targets was maintained at approximately 5 mm until the renal artery trunk was completely ablated (Figure 1). Post-ablation, angiography was performed to assess the condition of the renal arteries. All devices were then removed, and local pressure was applied to achieve hemostasis. Heparin (3000 U) was administered routinely during the procedure to prevent thrombus formation.

### Statistical analysis

Given the limited sample size and clinical heterogeneity, this study was designed as an exploratory descriptive case series. Therefore, results were mainly expressed using descriptive statistics and individual patient-level trajectories. Normally distributed data are presented as mean ± standard deviation (SD), while non-normally distributed data are summarized as median and interquartile range (IQR). Meanwhile, these tests were considered exploratory and were not used as the primary basis for inference. Results are primarily interpreted in terms of effect direction, magnitude of change, and consistency across individual patients rather than statistical significance. No formal adjustment for multiple comparisons was performed.

## Results

### Baseline characteristics

There are 10 patients enrolled in this study. Patient baseline characteristics are summarized in Table 1. All patients, with an average age of 57.9 ± 7.4 years, had refractory ES that required recurrent antitachycardia pacing (ATP) or ICD shocks. They underwent RFA with an average of 2.2 ± 0.4 times and received at least two antiarrhythmic medications before RDN. All patients were on long-term use of beta-blockers. Of the patients, 4 (40%) were diagnosed with ischemic cardiomyopathy (ICM), 6 (60%) had dilated cardiomyopathy (DCM), and 6 (60%) suffered hypertension. Patients had a left ventricular ejection fraction (EF) of 43.9 ± 12.5%, New York Heart Association Functional (NYHA) classification of 2.5 ± 0.7, NT-Pro BNP of 825.5 [91.1, 2822.5] ng/L, systolic blood pressure (SBP) of 109.9 ± 15.2 mmHg, and a diastolic blood pressure (DBP) of 66.5 ± 8.2 mmHg, LV-ESD of 50.5 ± 15.0 mm, LV-EDD of 64.2 ± 13.4 mm.

**Table 1:** Baseline patient characteristics.

Patient No.	Age (y)	Sex	BMI (kg/m^2^)	Etiology	NYHA class	NT-Pro BNP (ng/L)	EF (%)	Months of ICD	QRS Duration (ms)	No. of previous ablation procedures	Medications	Creatinine (*μ*mol/L)	SBP (mmHg)	DBP (mmHg)	Hypertension	LV-ESD (mm)	LV-EDD (mm)	NE (pg/mL)	E (pg/mL)
1	57	Male	29	ICM	3	2,660	46.56	54	118	2	A, BFT	78.19	115	65	Yes	48	63	-	-
2	52	Male	28	DCM	3	44.2	54.8	48	100	3	A, BB	88	122	79	Yes	44	62	204.1	30.72
3	59	Male	19.53	ICM	2	15,100	24.34	72	170	2	A, BB	114.89	89	58	Yes	82	93	-	-
4	63	Male	20	ICM	2	1,090	40.93	12	136	2	A, BFT	83.14	96	67	No	54	68	154.7	13.8
5	58	Male	26	ICM	2	97.1	59.88	12	100	2	A, BB	73.03	140	73	No	34	50	57.26	9.92
6	75	Male	24	DCM	2	3,310	48.9	30	98	2	A, BFT	99.17	121	75	Yes	43	57	149.8	11.27
7	60	Male	29	DCM	4	551	37.33	24	118	3	A, BB	76.84	95	51	No	49	60	172.4	17.29
8	51	Male	22	DCM	3	561	28.25	42	174	2	A, BB	63.61	101	66	Yes	69	80	72.12	3.4
9	55	Male	26	DCM	2	1,610	37.33	2	96	2	A, BB	99.7	112	62	Yes	49	60	431.4	79.66
10	49	Male	23	DCM	2	72.9	60.88	5	96	2	A, BB	73.5	108	69	No	33	49	-	-

ICM, ischemic cardiomyopathy; DCM, dilated cardiomyopathy; ES, electrical storm; LV-ESD, left ventricular end-systolic diameter; LV-EDD, left ventricular end-diastolic diameter; NE, norepinephrine; E, epinephrine; SBP, systolic blood pressure; DBP, diastolic blood pressure; A, amiodarone; BB, beta-blocker; BFT, bisoprolol fumarate tablets; Patient 1, The norepinephrine and epinephrine of patient 1, Patient 3 and Patient 10 are missing.

Unfortunately, three patients (Patient 1, Patient 3, and Patient 10) did not have

catecholamine test results available because of unavoidable factors. The other patients had an average norepinephrine level of 154.7 [72.1, 204.1] pg/mL and an average epinephrine level of 13.8 [9.9, 30.7] pg/mL upon admission.

### Procedure characteristic

The mean lengths of the right and left renal arteries were 46.5 ± 12.8 mm and 43.5 ± 13.3 mm, and the mean diameters of them were 5.0 ± 0.9 mm and 5.5 ± 1.0 mm separately (Table 2). The power of RFA was 17.0 ± 4.2 W, time duration was 24.0 ± 5.2 S, irrigation was 25.4 ± 5.1 mL/min and ablation lesions were 22.2 ± 10.2. One patient experienced temporary sluggish flow in right renal artery, while the other patients did not have any acute or chronic complications.

**Table 2:** RDN procedural characteristics.

Patient No.	Renal artery diameter (R/L)/mm	Renal artery length (R/L)/mm	No. of ablation lesions (R/ L)	Energy (W)	Duration (s)	Irrigation（mL/min）	Complications
P1	5.52/5.45	51.7/71.5	10/8	15	30	30	None
P2	5.91/7.46	42.7/42.1	11/12	25	30	30	None
P3	3.56/3.56	35.4/33.7	6/6	15	20	30	None
P4	5.84/6.11	40.4/57.4	11/12	25	30	30	None
P5	4.62/5.36	43.1/35.2	8/6	15	20	19	None
P6	5.32/5.80	68.5/48.1	20/20	15	20	20	None
P7	3.54/4.67	43.9/45.9	17/19	15	20	20	Sluggish flow in the right renal artery
P8	6.02/6.20	69.1/34.8	15/15	15	20	20	None
P9	5.45/4.74	35.6/41.6	5/7	15	30	30	None
P10	4.53/5.28	34.3/25.0	8/6	15	20	25	None

P1 to P10 represent patient 1 to patient 10.

### ICD therapies of pre-RDN and post-RDN

**Figure 2:**
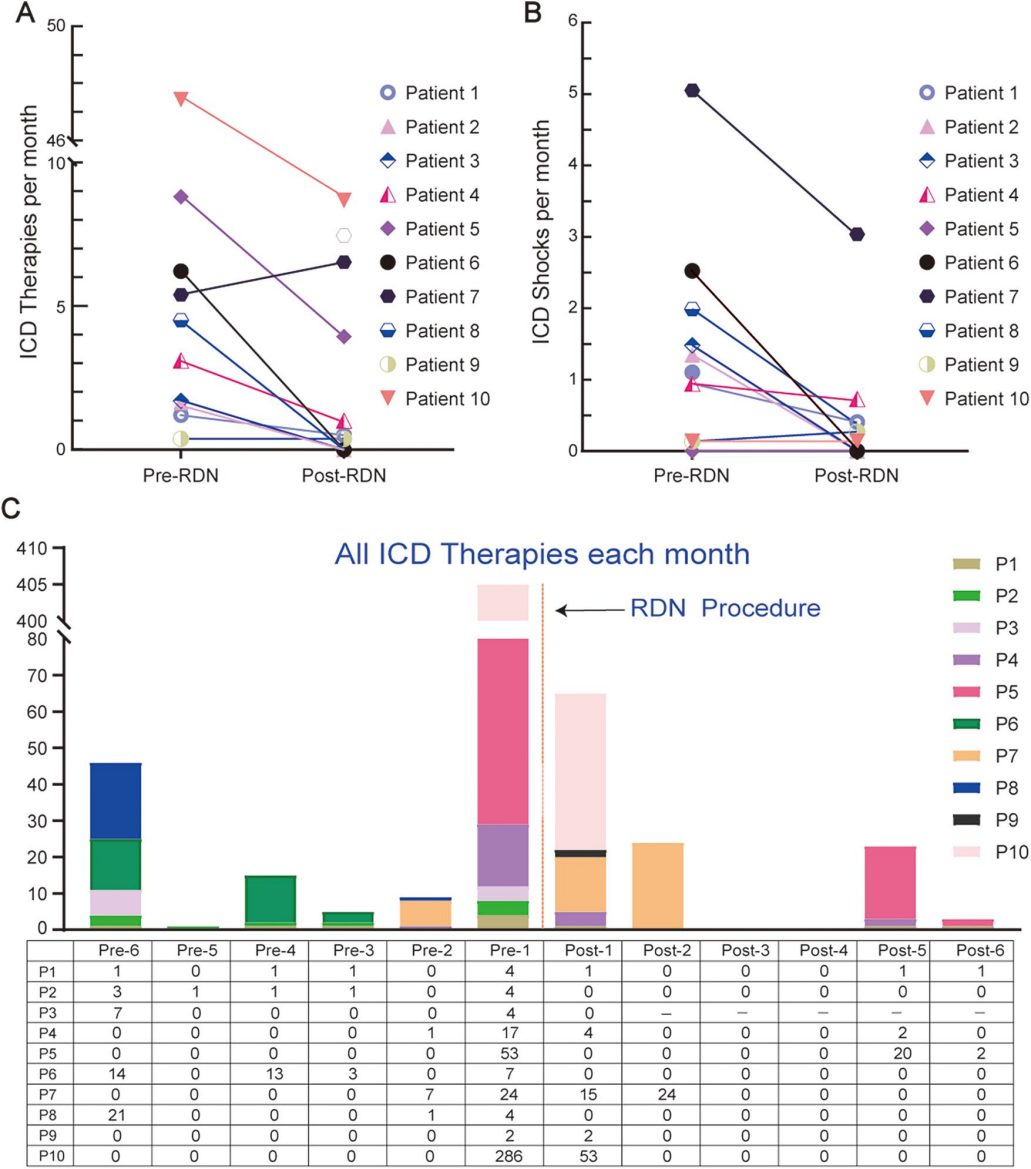
Details of ICD therapies of Pre- and posT,RDN. **(A)** All ICD therapies (shocks + ATP) in the 6-month follow-up. **(B)** ICD shock therapies in 6-month follow-up. P1 through P10 represent patients 1 through 10. Pre-1 to pre-6 represent 1 to 6 months before RDN, and post-1 to post-6 represent 1 to 6 months after RDN. ICD, implantable cardioverter-defibrillator, ATP, anti-tachycardia pacing, RDN, renal denervation, “-” means patient died.

The mean follow-up was 6 months with a range of 2 months to 6 months. One patient died 2 months after RDN, which was due to an accident according to his family. Interesting, we found a decreasing trend of ICD therapies per month after multimodal therapy including RDN [pre 3.0 [1.2, 6.2], post 0.3 [0.0, 1.0]]. Additionally, the ICD shock therapies experienced a similar tendency [pre 1.4 [0.2, 2.0], post 0.1 [0.0, 0.7]], as illustrated in Figure 2. One patient experienced frequent VAs on the postoperative day, which required repeated ATP therapies and was again combined with epicardial ablation on the following day, with 6-month follow-up results suggesting no ICD therapy. One patient experienced no decrease, and even a mild increase in all ICD therapies after RDN.

We then investigated the impact of the order in which RDN and RFA procedures are performed on the burden of treatments involving ICD. Among 10 patients, excluding 1 death, 5 of the remaining 9 patients underwent RFA simultaneously with RDN, while 4 patients underwent RDN treatment alone, with a mean interval of 8 ± 4.9 months since the last RFA.

### Cardiac and renal function

**Figure 3:**
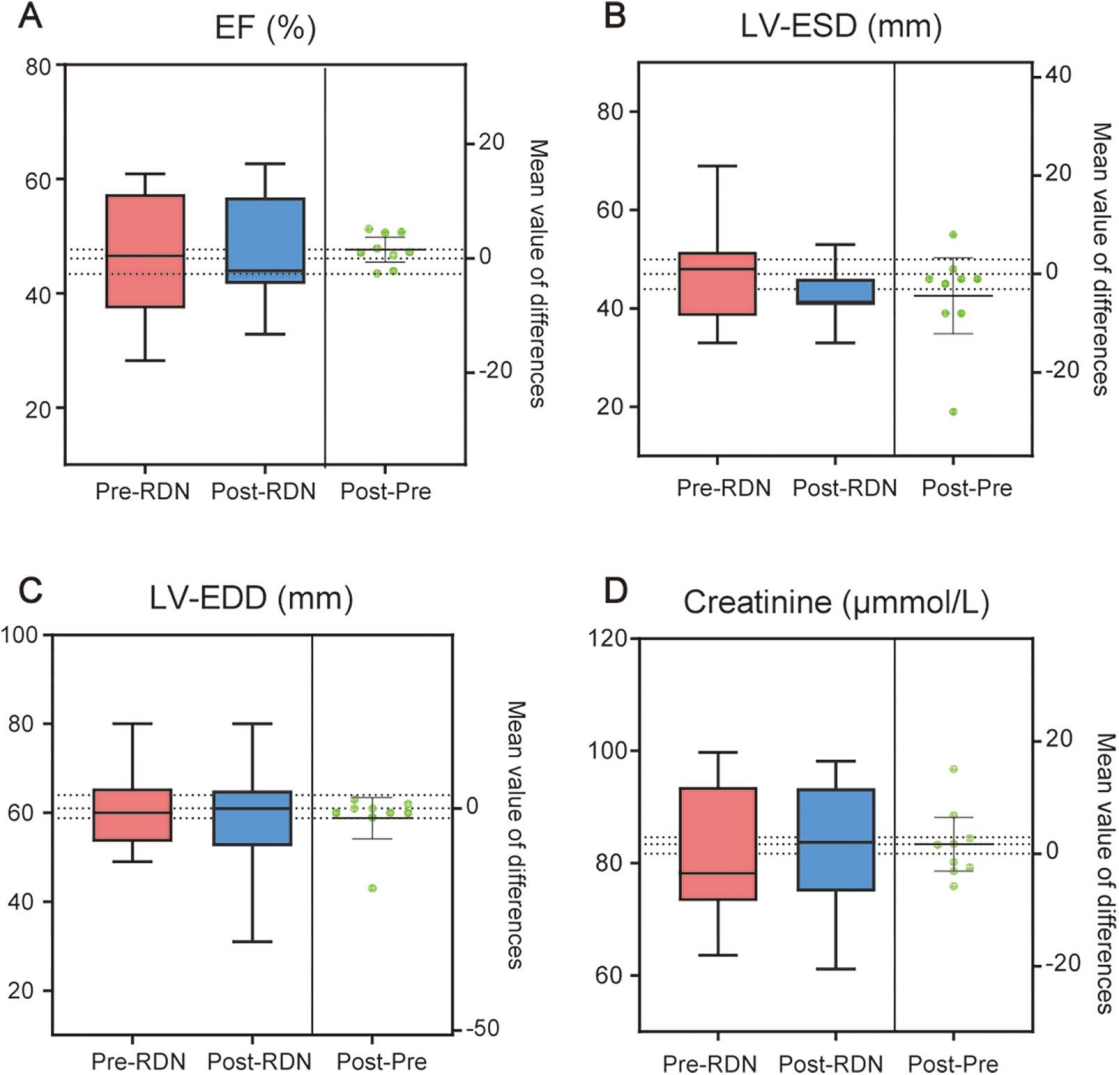
Echocardiography and creatinine at admission and 6 months post-RDN. The left side of each small graph represents the specific value of the indicator, and the right side represents the distribution of the difference between the two. **(A–D)** represents the change of pre-RDN and post-RDN; EF, ejection fraction; LV-ESD: left ventricular end-systolic diameter; LV-EDD: left ventricular end-diastolic diameter; RDN, renal denervation.

All patients underwent echocardiography and creatinine tests upon admission. During the 6-month follow-up, 1 patient passed away at the second month, and thus the analysis was conducted on the remaining 9 patients. The results showed that the average EF after RDN did not differ from the preoperative average (pre 47.6 ± 9.6%, post 46.1 ± 11.1%). Also, there was no obvious difference in left ventricular end-systolic diameter (LV-ESD) between preoperative and postoperative (pre 47.0 ± 10.8 mm, post 42.6 ± 5.6 mm) measurements. The left ventricular end-diastolic diameter (LV-EDD) also showed few changes from preoperative to postoperative (pre 61.0 ± 9.3 mm, post 58.8 ± 13.4 mm). Additionally, the average creatinine concentration postoperatively stayed unaffected (pre 83.4 ± 11.8 μmol/L, 81.7 ± 12.1 μmol/L), (Figure 3). Blood pressure measurements remained unchanged following RDN. Systolic blood pressure was unchanged after multimodal therapy RDN (pre 108.6 ± 15.7 mmHg, post 113.4 ± 11.5 mmHg), and diastolic blood pressure also remained not fluctuated (pre 65.1 ± 9.1 mmHg, post 68.0 ± 9.1 mmHg). Since 3 patients lacked measurements of norepinephrine and epinephrine, the catecholamine concentration of the remaining 7 patients was analyzed. Norepinephrine concentration of post-RDN exhibited a lowered tendency [pre 157.3 [149.8, 204.1] pg/mL, post 46.7 [34.6, 96.0]], while epinephrine concentration stayed stable [pre 16.4 [7.5, 29.8] pg/mL, post 13.8 [9.9, 30.7]]. In terms of drug treatment, except for P10 which increased the dosage, the remaining patients maintained the same or reduced the dosage (Table 3).

**Table 3:** Changes in medication before and after RDN.

Patient No.	Medications	Initial dose (mg)	Follow-up Dose (mg)
P1	A, Bis	200.00, 10.00	200.00, 5.00 (↓)
P2	A, BB	200.00, 95.00	200.00, 95.00
P4	Bis	15.00	10.00 (↓)
P5	A, BB	600.00, 95.00	200.00 (↓), 47.50
P6	A, Bis	600.00, 5.00	200.00 (↓), 5.00
P7	A, BB, Bis	200.00, 71.25, 0	200.00, 0, 5.00
P8	A, BB	600.00, 23.75	600.00, 23.75
P9	A, BB	600.00, 23.75	600.00, 23.75
P10	BB	47.50	95.00 (↑)

P1 to P10 represent patient 1 to patient 10. A, amiodarone; Bis, bisoprolol fumarate; BB, metoprolol succinate; Dron, dronedarone hydrochloride.

## Discussion

### Reduced ICD therapies and multimodal therapy including RDN

The retrospective, single-arm cohort study shows that in patients with ICDs and refractory ES, multimodal therapy including RDN might has the potential to reduce ICD therapies, while several factors are involved. First, 5 of 9 patients experienced RDN and RFA simultaneously, the decreasing trend of ICD therapies may be associated with RFA, the independent role of RDN is not guaranteed. Besides, one patient experienced one more epicardial ablation after RDN, even through the ICD therapy stopped, the reduced ICD therapy still correlated with ablation. Moreover, the antiarrhythmic drug dosage increased after RDN procedure in one patient, the reduction of ICD therapies might be related to drug intervention. Hence, the lowered trend of ICD therapy might be the integrated role of RDN, RFA and antiarrhythmic drugs. One purpose of our current study is to evaluate the potential effectiveness of RDN, while based on current limited sample size, the effectiveness can only be interpretated as the combined effect of multimodal therapy, further investigation with larger sample size may help to validate the hypothesis of effectiveness.

### Correlation between reduced catecholamine levels and reduction of ICD therapies after multimodal therapy including RDN procedure

On dog myocardial infarction (MI) models, our center has previously revealed that reduced catecholamine levels and sympathetic remodeling was the underlying mechanism of RDN, it can prevent cardiac remodeling and ventricular arrhythmias. Other studies have also shown that sympathetic excitation plays a certain role in patients with VAs. VAs may be related to enhanced automaticity, triggered automaticity, and reentrant mechanisms; all three mechanisms are markedly potentiated by the action of catecholamines (7), and CSD can reduce the burden of VAs in patients, which demonstrated the role of the sympathetic nervous system in VAs (9). Interestingly, even in patients who have already completed CSD, RDN can still reduce the VTs burden, and the results of the staged procedure are even better, indicating that the RDN on VTs is not limited to cardiac nerves, and may be related to its reduction of catecholamine concentration in the blood (10). Even through consistent with former studies, 7 of our patients experienced a reduced tendency of norepinephrine level, epinephrine remained unchanged after RDN, and 3 patients lack the related plasma catecholamine results. Taken together, the restricted sample number limited our statistical power, and we therefore cannot ensure that the catecholamine changes were the independent role of RDN, or reduced sympathetic activity prevent the ICD therapies. However, we can still hold the hypothesis that sympathetic modulation might be associated with reduced ICD therapies, and we believe larger sample size is needed to further clarify and validate the underlying mechanism.

### One patient without reduction of ICD therapy after RDN

9 of 10 patients experienced a reduced trend of ICD therapies after RDN intervention combined with RFA and antiarrhythmic intervention, while one patient experienced very high-frequency ventricular electrical storms before admission. To promise his safety with our greatest effort, RDN combined with epicardial ablation was performed, but the patient still had frequent VT the next day. After intensified drug treatment, the patient's VT was significantly reduced. Later follow-up showed no ICD therapy events. For this patient with decreasing ICD therapies after multiple interventions, we still cannot distinguish the “effective” role is attribute to RDN, RFA or intensified antiarrhythmic intervention.

### Changed catecholamine levels and variable function of sympathetic nerves

Overall, the reduction of shock therapies in post-RDN patients might be correlated with reduced catecholamines and sympathetic nerves as our hypothesis. Based on the speculation and current studies, the potential mechanism may be that RDN impairs the sympathetic network and blocks the nerves of the renal artery, including afferent and efferent nerves. On the one hand, RDN can block afferent nerves and reduce central nervous system stimulation and subsequently decrease cardiac sympathetic nerve excitation. Previous studies have also confirmed that afferent nerves can reduce stimulation of central nervous system nuclei and simultaneously reduce the excitability of peripheral organ sympathetic nerves (3–15). In this study, we assessed the skin sympathetic nerve activity before and after RDN by PowerLab (Bio Amp; AD Instruments) and found a relative reduction in sympathetic activity, reflecting the potential impact of multimodal therapy (including RDN) on the systemic sympathetic nervous system (Supplementary Figure 1). This result also aligns with our previous animal studies (8). Taken together, we still cannot draw the conclusion that the sympathetic nerves were affected or the changed catecholamine levels were correlated with RDN intervention, we hope mechanistic studies of RDN on patients with ES may further clarify the potential connections and therapeutic targets.

### RDN for the therapy of VAs and ES

RDN has been explored as an adjunctive neuromodulatory therapy for refractory VAs, based on the role of sympathetic overactivation in arrhythmogenesis. Early case series and observational studies suggested that RDN may reduce ventricular fibrillation episodes and implantable cardioverter-defibrillator therapies in patients with structural heart disease who remained symptomatic despite antiarrhythmic drugs and catheter ablation (1–3). However, current evidence is still limited with restricted sample sizes, heterogeneous populations, and non-randomized study designs. Recent systematic reviews indicate a potential reduction in VA burden after RDN, but its efficacy and safety require confirmation in larger controlled trials (1, 2). Since RDN seems effective in patients with VAs, which drive us to further explored its role in ES. Similar with our current results, multimodal therapy with RDN may have the potential to influence ICD therapies in patients with ES, while it remains unclear to judge the contribution ratio, the RDN might be effective, the RFA and antiarrhythmic drugs may also be therapeutic. Therefore, in patients with ES, larger sample size studies may further to be conducted to validate the hypothesis.

### Current status and future prospect

In recent years, renal nerve stimulation (RNS) for locating and ablating the target site has provided a standardized reference for precise RDN. Previous studies have suggested that a post-RNS SBP rise > 10 mmHg indicates a site rich in sympathetic nerves, and ablation at this site will yield favorable results (16, 17). Meanwhile, a recent meta-analysis evaluated the effectiveness of RDN in patients with HF with reduced ejection fraction (EF). Including 8 prospective RCT studies, RDN can improve left ventricular systolic function and increase EF, but there was no effect on LV-ESD (18). This study also evaluated changes in left ventricular function and cardiac structure in patients after RDN. However, the results showed no improvements of EF, LV-ESD, and LV-EDD. Possible reasons include the poor initial cardiac structure and function of the admitted patients, making significant improvement more difficult or slower than in other patients; secondly, the overall follow-up period was short, preventing observation of significant changes. Therefore, more standardized surgical procedures and longer post-operative follow-ups are needed to evaluate the effectiveness and safety of RDN.

## Conclusion

Multimodal therapy including RDN may has the potential to contribute to decrease ICD therapies for patients with ES. Larger sample investigations may further demonstrate the efficacy and safety of RDN.

## Limitation

This is a retrospective analysis of data from a single center including only 10 patients, statistical power may be limited. The relevant description of results and discussion on RDN effectiveness can only express the correlation for RFA and drug intervention were also integrated, further validation must depend on studies with larger sample size. Before RDN procedure, patients have experience RFA or antiarrhythmic drug therapy, based on such confounding factors, we cannot realize isolation of the independent effects of RDN, the result of ICD reduction cannot only attribute to RDN. RDN can only be treated as an adjunctive method for patients with ES in multimodal therapy. For one unexpected death during the follow-up after RDN, we thus cannot promise that RDN is totally safe even through the cardiac function is poor and the echocardiography and plasma creatinine of all the other 9 patients remained unchanged. Catecholamine results of 3 patients are missing and the epinephrine levels remain unchanged. Meanwhile, catecholamine level measuring cannot realize autonomic assessment, so the “therapeutic” effect of RDN with other interventions may only be related with reduced catecholamine, but not directly. Short-term of follow-up cannot reflect cardiac remodeling after RDN, longer follow-up and larger sample sizes are needed for validation. Since multiple parameters were included in our study, with restricted sample size, multiple comparison is difficult to achieve. Although the same physician performed RDN in all cases, which minimizes the impact on the results, we did not conduct RNS; instead, we used anatomical localization for ablation. Additionally, the limited sample size and short follow-up period may introduce bias into the results. We will address these limitations in our upcoming prospective multicenter randomized controlled trial. We would also perform preoperative and postoperative skin sympathetic nerve activity testing on all patients for a more comprehensive assessment of the impact of RDN, avoiding exaggeration or underestimation of its efficacy.

## Data Availability

The raw data supporting the conclusions of this article will be made available by the authors, without undue reservation.
