# Supplementary material for: Efficacy and safety of renal denervation in patients with refractory ventricular electrical storm
Source: Front Cardiovasc Med. 2026 Jul 28;13:1840013. doi: 10.3389/fcvm.2026.1840013 (PMC13457448; doi:10.3389/fcvm.2026.1840013)
Supplement: Supplementary file 1 [file Image1.pdf]

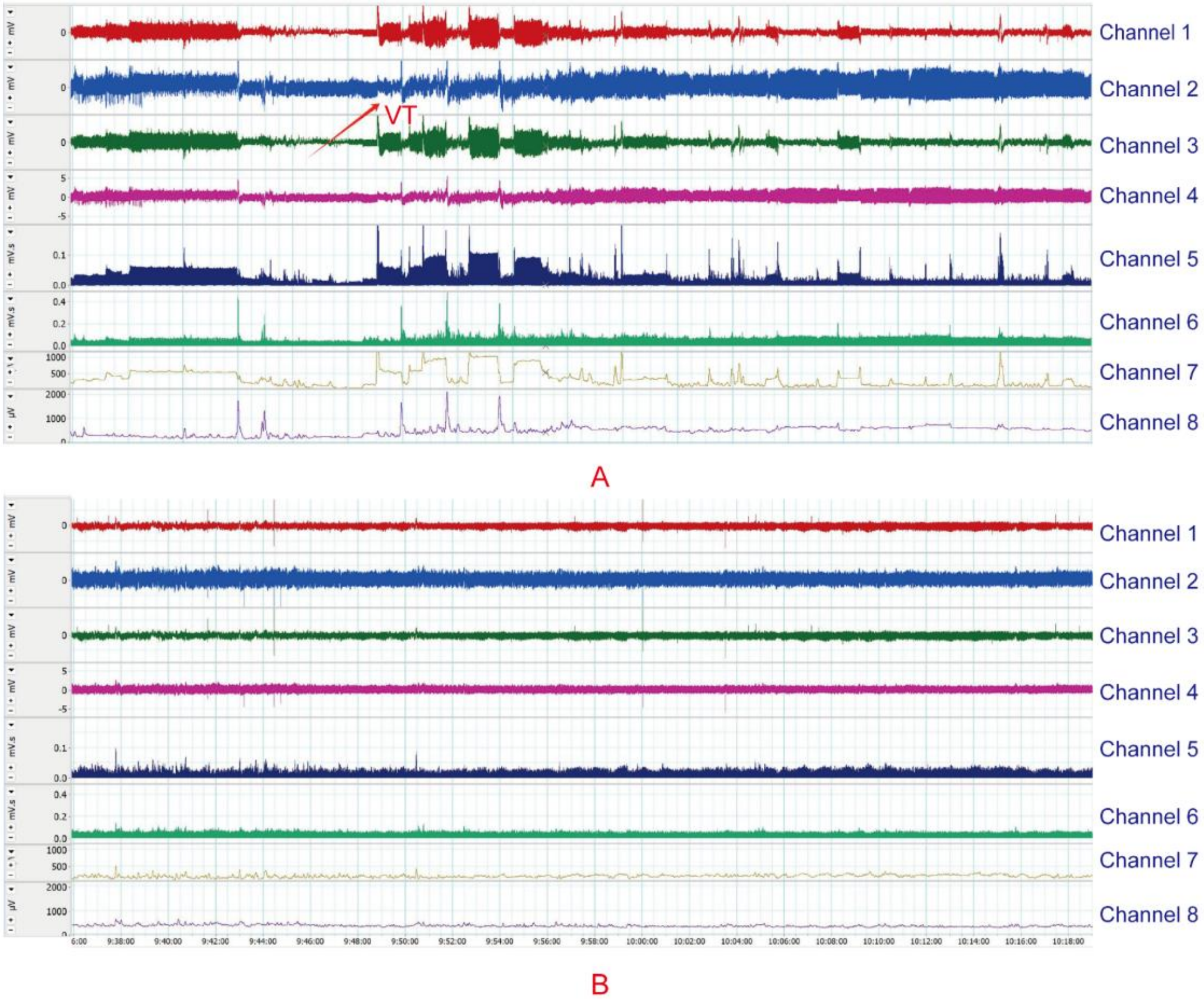

Supplementary Figure 1. Recordings of skin sympathetic nerve activity before and after surgery. (A) Preoperative sympathetic nerve recording results; (B) Postoperative sympathetic nerve recording results; Channel 1 and 2 capture electrical signals ranging from 1 to 20,000 Hz, representing the overall signal; Channel 3 and 4 capture electrical signals between 500 to 1,000 Hz, indicating skin sympathetic nerve activity; Channels 5 and 6 employ an integral measurement technique to sample the absolute value every 0.1 seconds, which indicates integrated skin sympathetic nerve activity; Channels 7 and 8 apply this technique to average the absolute values of five samples each second, representing average skin sympathetic nerve activity; The amplitude of the waveform represents the strength of the recorded electrical signal.
